# Surgical and medical emergencies on board European aircraft: a retrospective study of 10189 cases

**DOI:** 10.1186/cc7690

**Published:** 2009-01-20

**Authors:** Michael Sand, Falk-Georges Bechara, Daniel Sand, Benno Mann

**Affiliations:** 1 Department of General and Visceral Surgery, Augusta Krankenanstalt, Academic Teaching Hospital of the Ruhr-University Bochum, Bergstrasse 26, 44791 Bochum, Germany; 2 Department of Dermatology and Allergology, Ruhr-University Bochum, Klinikstrasse 56, 44791 Bochum, Germany; 3 Department of Physiological Science, University of California Los Angeles (UCLA), 621 Charles E. Young Drive South, Los Angeles, CA 90095-1527, USA

## Abstract

**Introduction:**

In-flight medical and surgical emergencies (IMEs) onboard commercial aircrafts occur quite commonly. However, little epidemiological research exists concerning these incidents.

**Methods:**

Thirty-two European airlines were asked to provide anonymous data on medical flight reports of IMEs for the years 2002 to 2007. The total number of incidents was correlated to revenue passenger kilometers (rpk). Additionally, on-board births and deaths, flight diversions, flight routes (continental/intercontinental) and involvement of a physician or medical professional in providing therapy were analysed.

**Results:**

Only four airlines, of which two participated in this study, were able to provide the necessary data. A total of 10,189 cases of IMEs were analysed. Syncope was the most common medical condition reported (5307 cases, 53.5%) followed by gastrointestinal disorders (926 cases, 8.9%) and cardiac conditions (509 cases, 4.9%). The most common surgical conditions were thrombosis (47 cases, 0.5%) and appendicitis (27 cases, 0.25%). In 2.8% of all IMEs, an aircraft diversion was performed. In 86% of cases, a physician or medical professional was involved in providing therapy. A mean (standard deviation) of 14 (+/- 2.3, 10.8 to 16.6 interquartile range) IMEs per billion rpk was calculated.

**Conclusions:**

The study demonstrates that although aviation is regulated by a variety of national and international laws, standardised documentation of IMEs is inadequate and needs further development.

## Introduction

As aircraft passenger load increases, presently exceeding 40 million passengers per year worldwide, in-flight surgical and medical emergencies (IMEs) on commercial aircrafts also occur quite frequently. A variety of low-cost carriers have made air-travel accessible to a larger portion of the population, contributing to increasing passenger load. Additionally, the average passenger age is also steadily rising because of increased life expectancy in western countries. It has been estimated that by the year 2030, half of all aircraft passengers will be over 50 years of age [1]. In addition to the continuous increase in the average age of passengers, flight stress and changes in the cabin environment (temperature, humidity or air pressure), and other additional factors associated with travel, such as the stress of increased security, decreased seat space and increasing delays, can also trigger medical emergencies on board [1].

Although some airlines make an effort to document IMEs as precisely as possible, there is still a lack of standardisation, resulting in a variety of data, which hampers epidemiological research on IMEs. This may be a result of the void in legal obligation for airlines to monitor and report IMEs. However, this epidemiological research is necessary to adapt and standardise the contents of medical flight kits (MFK) on airplanes, which have a considerable variability both in medication and equipment [2]. Furthermore, it would be useful to improve preventive strategies in assisting pre-flight medical screening of patients [3]. Recent data on IMEs is sparse, often based on a single airline and a short time period, and is not correlated to revenue passenger kilometers (rpk), which does not allow for an objective analysis [4,5].

In the present study, all documented IMEs of two European airline carriers between the years 2002 and 2007 were included. The medical flight report statistics provided by each individual airline were subjected to a descriptive analysis, which included the frequency and type of emergency. The frequency of aircraft diversion was also investigated. All data regarding names, ages and the sex of the patient and the name of the airline were anonymous. The goal of this retrospective study was to document medically relevant emergencies in airline passengers from 2002 to 2007 on board European aircraft.

## Materials and methods

This study originates from the surgical department of an academic teaching hospital (Department of General and Visceral Surgery, Augusta Krankenanstalt, Academic Teaching Hospital of the Ruhr-University Bochum). A total of 32 European airlines were asked to provide data on IMEs. All patients between January 2002 and December 2007 were included in the study. The following data were also recorded: on-board births and deaths, flight diversions, whether the incident occurred on a continental or intercontinental flight, and the involvement of a physician or medical professional (nurse or paramedic) in providing therapy.

The authors retrospectively reviewed the available data and classified different categories of medical and surgical emergencies. Only events that actually happened in the air after take-off and before landing were included. rpk values were also obtained from the individual airlines. rpk is a measure of the volume of passengers carried by an airline; it is the sum of the products obtained by multiplying the number of revenue passengers carried on each flight by the distance. It describes the total number of kilometres travelled by all passengers and therefore objectifies data analysis. It is regularly used in commercial aviation to report the sales volume of passenger traffic. In order to objectify data, total emergencies per year were related to the airlines' total rpk.

While handling the data, the regulations of the Ethic commission of the Ruhr-University Bochum were fully respected (ClinicalTrials.gov Identifier: NCT00713102, Ethical Review Board of the Ruhr-University Bochum, Germany, registration number: 3207-08). As noted, evaluation of the data was performed anonymously without any information regarding the airline or no other passenger details except their illness. Institutional Review Board approval was obtained and informed consent was waived. The collected data were compiled in an electronic database (Microsoft Excel for Windows, Microsoft Corp., Redmond, WA), mean values for numeric items were calculated and the resulting data were evaluated.

## Results

Of a total of 32 European airlines included in the study, only four were able to provide the required data with adequate medical flight reports. Two of these did not participate in the study due to company policy. One airline was able to provide data but did not qualify for inclusion as the provided diagnoses of the patients were not specific enough to be included in the study. Twenty-seven airlines were not able to provide the necessary data for inclusion in the study. After inspection of all available data, a total of 10,189 patients with an IME on board two European airlines were enrolled in the study. Data were provided from one airline for the years 2002 to 2007 and from another for 2006 to 2007. The total rpk analysed in this study included a total of 613.03 billion rpk.

Of all emergencies documented, 20.4% were on continental flights and 79.6% were on intercontinental flights. A total of 279 diversions occurred among the 10,189 in-flight patients (2.8%). In the year 2007, 58% of the diversions were on intercontinental flights and 42% on continental flights. A physician was on board in 77.4% of the diversions. The most frequent causes for diversion were myocardial infarction (22.7%), apoplexy (11.3%) and epileptic seizures (9.4%). In 86% of the emergencies between 2002 and 2005, a physician or medical professional (nurse or emergency medical technician) was involved in on-board patient therapy. Data regarding physician involvement, except for diversions, were not available for the years 2005 to 2007.

Based on a total of 10,189 emergencies analysed here, an average mean (standard deviation) of 14 (± 2.3, 10.8 to 16.6 interquartile range) emergencies per billion rpk were calculated.

Aircraft diversion was performed in 279 cases (2.8%) (Table 1). Syncope was by far the most common medical condition reported (5307 cases, 53.5%). Gastrointestinal disorders were responsible for 8.9% of all emergencies (926 cases). The third most common medical emergency was cardiac conditions (509 cases, 4.9%), followed by fear of flying (460 cases, 4.3%) and generalised pain (432 cases, 4.1%). Details of all diagnoses are summarised in Table 2.

**Table 1:** Annual emergencies per billion revenue passenger kilometres (rpk) and flight diversions.

**Year**	**2002**	**2003**	**2004**	**2005**	**2006**	**2007**
Emergencies/billion rpk	16.6	13.4	10.8	15.8	15.4	11.8

Aircraft diversions	26	41	47	44	55	66

**Table 2:** Details of medical and surgical in-flight emergencies. Percentages are based on 10,189 incidents from two European airlines January 2002 to December 2007.

**Year**	2002		2003		2004		2005		2006		2007	
	n	%	n	%	n	%	n	%	n	%	n	%
	
**Diagnosis**	1615	100%	1210	100%	1167	100%	1692	100%	2379	100%	2126	100%

**Syncope**	906	56.1%	665	55.0%	726	62.2%	919	54.3%	1028	43.2%	1063	50.0%
**Gastrointestinal disorders**	150	9.3%	89	7.4%	91	7.8%	160	9.5%	253	10.6%	183	8.6%
**Generalised pain**	89	5.5%	50	4.1%	29	2.5%	63	3.7%	100	4.2%	101	4.8%
**Fear of flying, unruliness**	73	4.5%	42	3.5%	33	2.8%	91	5.4%	118	5.0%	103	4.8%
**Cardiac condition**	64	4.0%	52	4.3%	58	5.0%	93	5.5%	148	6.2%	93	4.4%
**Nausea and vomiting**	52	3.2%	23	1.9%	30	2.6%	49	2.9%	87	3.7%	58	2.7%
**Allergy**	37	2.3%	42	3.5%	24	2.1%	25	1.5%	40	1.7%	54	2.5%
**Pyrexia**	30	1.9%	35	2.9%	18	1.5%	26	1.5%	50	2.1%	30	1.4%
**Accident**	26	1.6%	22	1.8%	18	1.5%	46	2.7%	165	6.9%	82	3.9%
**Hypoglycaemia**	23	1.4%	30	2.5%	8	0.7%	16	0.9%	14	0.6%	12	0.6%
**Renal colic**	22	1.4%	27	2.2%	10	0.9%	16	0.9%	22	0.9%	17	0.8%
**Epileptic seizure**	19	1.2%	36	3.0%	28	2.4%	31	1.8%	61	2.6%	44	2.1%
**Dyspnoea**	18	1.1%	5	0.4%	2	0.2%	3	0.2%	4	0.2%	2	0.1%
**Asthma, dyspnoea**	14	0.9%	7	0.6%	8	0.7%	22	1.3%	65	2.7%	68	3.2%
**Inebriation**	13	0.8%	6	0.5%	5	0.4%	4	0.2%	11	0.5%	11	0.5%
**Thrombosis**	9	0.6%	8	0.7%	6	0.5%	11	0.7%	8	0.3%	5	0.2%
**Biliary colic**	9	0.6%	4	0.3%	5	0.4%	4	0.2%	9	0.4%	2	0.1%
**Migraine**	8	0.5%	4	0.3%	2	0.2%	4	0.2%	2	0.1%	8	0.4%
**Epistaxis**	8	0.5%	2	0.2%	5	0.4%	8	0.5%	7	0.3%	5	0.2%
**Deaths**	6	0.4%	3	0.2%	5	0.4%	5	0.3%	13	0.5%	20	0.9%
**Hyperventilation**	6	0.4%	8	0.7%	2	0.2%	9	0.5%	27	1.1%	13	0.6%
**Appendicits**	6	0.4%	3	0.2%	3	0.3%	5	0.3%	4	0.2%	6	0.3%
**Pregnancy problems**	6	0.4%	4	0.3%	5	0.4%	13	0.8%	7	0.3%	8	0.4%
**Diabetes**	4	0.2%	7	0.6%	19	1.6%	26	1.5%	45	1.9%	34	1.6%
**Suspected malaria**	4	0.2%	0	0.0%	1	0.1%	1	0.1%	0	0.0%	1	0.0%
**Suspected apoplexy**	4	0.2%	9	0.7%	6	0.5%	16	0.9%	14	0.6%	17	0.8%
**Suspected MI**	4	0.2%	2	0.2%	6	0.5%	2	0.1%	10	0.4%	10	0.5%
**Hypertension**	2	0.1%	12	1.0%	10	0.9%	15	0.9%	55	2.3%	39	1.8%
**Narcotic substance abuse**	1	0.1%	1	0.1%	1	0.1%	2	0.1%	2	0.1%	1	0.0%
**Suspected meningitis**	1	0.1%	0	0.0%	0	0.0%	0	0.0%	0	0.0%	0	0.0%
**Labor pains**	1	0.1%	5	0.4%	2	0.2%	0	0.0%	1	0.0%	8	0.4%
**Births**	0	0.0%	0	0.0%	0	0.0%	0	0.0%	0	0.0%	2	0.1%
**Suspected embolism**	0	0.0%	1	0.1%	0	0.0%	2	0.1%	2	0.1%	1	0.0%
**Suspected pneumonia**	0	0.0%	3	0.2%	0	0.0%	1	0.1%	0	0.0%	1	0.0%
**Drug abuse**	0	0.0%	0	0.0%	0	0.0%	3	0.2%	6	0.3%	23	1.1%
**Suspected tuberculosis**	0	0.0%	1	0.1%	0	0.0%	1	0.1%	0	0.0%	0	0.0%
**Aneurysm**	0	0.0%	1	0.1%	0	0.0%	0	0.0%	0	0.0%	0	0.0%
**Gastrointestinal bleeding**	0	0.0%	1	0.1%	0	0.0%	0	0.0%	0	0.0%	0	0.0%
**Cerebral haemorrhage**	0	0.0%	0	0.0%	1	0.1%	0	0.0%	1	0.0%	0	0.0%
**Attempted suicide**	0	0.0%	0	0.0%	0	0.0%	0	0.0%	0	0.0%	1	0.0%

Surgical illnesses accounted for a minor percentage of all on-board emergencies. Thrombosis (47 cases, 0.5%), appendicitis (27 cases, 0.25%) and gastrointestinal bleeding (1 case, < 0.1%) were categorised as surgical emergencies. There were two births (< 0.1%) and 52 deaths (0.5%) in our study. After analysing the emergencies per rpk, we could not detect an increase in incidence of IMEs over the years 2002 to 2007. The details of these findings are summarised in Table 1.

## Discussion

Although IMEs are generally rare, they can have a significant effect on other passengers and crew, potentially with operational implications for the flight [6]. Their incidence has been reported to be one per 10 to 40,000 passengers, with more than a total of two billion passengers travelling on commercial airlines each year [7,8]. In order to make the data objective and comparable, we presented it in relation to rpks. We calculated an average mean of 14 (± 2.3, 10.8 to 16.6) emergencies per billion rpk for the 10,189 emergencies analysed.

In contrast to recent studies, which suggest that the frequency of IMEs is increasing, based on our analysis from 2002 to 2007, we were unable to confirm this observation [9]. However, our analysis should be interpreted with restraint, as not every medical incident is appropriately documented and, further, this study is not comprehensive, as only two airlines contributed the analysed data.

Analysing the available data, the breakdown of the various medical emergencies encountered in our study showed that syncope was by far the most frequent medical condition (5307 cases, 53.5%), followed by gastrointestinal disorders (926 cases, 8.9%) and cardiac conditions (509 cases, 4.9%), which are similar results to those seen in other studies [10,11]. One major problem that we encountered was a lack of standardisation in terms of diagnostic categorisation and confirmed diagnostic data. This was reflected in the fact that only four out of 32 airlines were able to contribute to the study, only two of which could ultimately be enroled. Worldwide, it has been reported that only 17% of all IMEs are documented, most of them inconsistently, which would seem to indicate that legislation for mandatory standardised documentation and the establishment of an international registry is needed [12].

Flying on commercial aircrafts has been identified as the safest form of travel. Nevertheless, the special environment in an airplane constitutes a physiological and psychological stressor for many individuals, potentially triggering a variety of medical emergencies that may occur on board. This can lead to challenging situations for physicians offering help. Based on ethical and legal duties, every physician is required to offer help within his or her scope of practice. The legal duty, however, is only applicable for certain countries. In the USA, Canada and the UK physicians on airplanes are not required by law to respond to a call for help [8]. In contrast, the European Union and Australia require physicians on board to do so.

Physicians helping in IMEs on board airplanes are protected by the so-called Good Samaritan Act [13]. For airlines registered in the USA, the Medical Assistance Act of 1998 additionally protects physicians who provide medical help from possible legal consequences. Furthermore, the Tokyo Convention Act of 1963 allows passengers to take actions which are necessary to prevent disruptive passengers from endangering the safety of the flight [14]. Other regulations that touch on IMEs differ depending on the origin of the aircraft. For example, in the USA, the US Federal Aviation Administration (FAA) requires every US registered commercial aircraft with more than one flight attendant or 12 seats to carry an automatic external defibrillator (AED). Although most large national European national airlines carry AEDs, some of them only do so for intercontinental flights. Unfortunately, there is no law that mandates that an AED must be included in the MFK for commercial aircrafts registered in Europe.

The MFK contents in European commercial aircrafts are not precisely regulated, which results in a variety of different medications and equipment on board. In Germany, the regulations of the National Federal Aviation Agency (Luftfahrt-Bundesamt, Braunschweig, Germany) and the European Joint Aviation Authorities (JAA; Cologne, Germany) regulate aviation on the national and continental level. They regulate by law the contents of an on-board dispensary and the MFK. However, in Europe, the regulations regarding equipment and medication are loosely formulated, giving airlines broad flexibility in assembling their MFKs while adhering to the law [15,16]. Now more than ever, cost-cutting pressures on airlines make it unsurprising that the contents of on-board medical kits differ considerably.

The first author (MS) had the opportunity to compare the MFK of a large national European national airline with that of a low-cost (no-frills) carrier. Although the national European airline had excellent equipment, intravenous medications and an AED on board, the MFK of the low-cost carrier showed only basic equipment without any intravenous medication or indwelling venous canulas, which could be of importance if reanimation is needed. Although this is a single experience with one airline, we feel that we can assume similar discrepancies in comparable airlines. Therefore, it would seem advisable for some airlines, despite the economic pressure, to reassess their MFKs with regard to their responsibilities to passengers' safety.

Several studies have shown the use and suitability of expanded mandatory medical kits introduced on board of US airlines in 1996, which caused the US Federal Aviation Administration (FAA) to prescribe that an emergency kit with intravenous drugs, AED and other advanced emergency equipment must be on board [17]. The Air Transport Medicine Committee of the Aerospace Medical Association is continuing to work on and publish recommendations for MFK contents [18]. Considering the fact that cardiac conditions were the third most common condition seen in this study (509 cases, 4.9%), patients with cardiac irregularities may profit from an on-board AED as part of the MFK. The same is true for patients with a suspected myocardial infarction (34 cases, 0.3%). Apart from passengers who would benefit from an expanded MFK, flight crew members can also be affected by a medical incident on board, especially as there are special health risks associated with being an airline crew member [19,20]. Between 1968 and 1988, Air France reported 10 pilots were incapacitated by cardiac arrhythmias, seizures and hypoglycaemia during flight [8]. In one incident, carbon dioxide from improperly packed dry ice was the reason for the incapacitation of an entire cockpit crew [21].

The rate of aircraft diversion in our study was 2.8% (279 diversions). Other studies report diversion rates of 13% and 7.9%, whereas Cathay Pacific reported 0.35% for the year 2005 [10,22]. Besides its important medical impact, IMEs leading to aircraft diversion also have a considerable economic and ecological impact. A fully loaded Boeing 747 needs 23.5 litres kerosene/100 km at the start phase on the ground, which is about 2 km long and 3.4 litres kerosene/100 km on the climb flight, which is about 100 km. In cases of flight diversion, the impact of dumping fuel due to weight restrictions for landing is an additional financial and ecological factor. Besides the logistical challenge, aircraft diversion is also accompanied by a significant financial loss. The total costs of a diversion depend on the size of the aircraft, ranging from $30,000 to $725,000 per diversion, which may encourage airlines to focus on improved pre-flight screening of chronically ill patients [3,10,23].

## Conclusions

A standardised epidemiological database documenting IMEs on-board commercial aircrafts will provide access to potentially valuable data for further flight-epidemiological research. However, standardisation of IME reporting is necessary for further larger studies to be conducted, as the current quality of data is poor.

## Key messages

• An analysis of 10,189 medical flight reports revealed syncope (53.5%), gastrointestinal disorders (8.9%), cardiac conditions (4.9%), fear of flying (4.3%) and generalised pain (4.1%) as being the five most frequent diagnoses.

• The most frequent diagnosis causing flight diversion were myocardial infarction (22.7%), apoplexy (11.3%) and epileptic seizures (9.4%).

• Standardised documentation of IMEs is inadequate and needs further development. An international registry could assist future studies

## Abbreviations

AED: automatic external defibrillator; FAA: US Federal Aviation Administration; IME: in-flight medical and surgical emergency; MFK: medical flight kits; rpk: revenue passenger kilometres.

## Competing interests

The authors declare that they have no competing interests.

## Authors' contributions

MS participated in the study design, data analysis and interpretation of the data as well as the writing of the manuscript. FGB participated in the data analysis and interpretation of the study. DS participated in the data analysis, literature search, revision of the bibliography, the revision and editing of most of the manuscript. BM participated in the data analysis, the revision and editing of part of the manuscript. MS, FGB, DS and BM critically revised the manuscript for intellectual content. All authors read and approved the final manuscript.
